# Inelastic electron scattering at a single-beam structured light wave

**DOI:** 10.1038/s42005-023-01300-2

**Published:** 2023-07-15

**Authors:** Sven Ebel, Nahid Talebi

**Affiliations:** 1 Institute of Experimental and Applied Physics, Kiel University, Kiel, Germany; 2 Kiel Nano, Surface and Interface Science KiNSIS, Kiel University, Kiel, Germany

**Keywords:** Matter waves and particle beams, Ultrafast photonics

## Abstract

In free space, electrons undergo inelastic scattering in the presence of ponderomotive potentials generated by light pulses and standing light waves. The resulting modulated electron energy spectrum can exhibit the formation of discrete energy sidebands when multiple light beams are employed. Here, we demonstrate the inelastic scattering of slow-electron wavepackets at a propagating Hermite-Gaussian light beam. The pulsed Hermite-Gaussian beam thus forms a ponderomotive potential for the electron with sufficient momentum components, leading to the inelastic scattering and subsequent formation of discrete energy sidebands. We show that the resulting energy-gain spectra after the interaction are strongly influenced by the self-interference of the electrons in this ponderomotive potential. This effect is observable across various wavelengths, and the energy modulation can be controlled by varying the electron velocity and light intensity. By utilizing the vast landscape of structured electromagnetic fields, this effect introduces an additional platform for manipulating electron wavepackets.

## Abstract (web summary)

Inelastic interactions between electrons and light occur in free space through the presence of ponderomotive potentials generated by light. Here, the authors demonstrate that the use of a single spatially structured traveling light beam modulates the final electron energy spectrum, exhibiting the formation of discrete energy sidebands.

## Introduction

The scattering of electrons^1–3^, atoms^4^, and molecules^5^ at light has been the subject of intensive research activities for many decades. All these works are based on the prediction made by Kapitza and Dirac^1^ that the matter wave is diffracted from a grating that is formed by two counterpropagating light waves. The thereby acting physics can be understood equivalently either from a particle or matter-wave point of view^6^. In the particle picture, a photon is absorbed by the electron and a second photon is emitted simultaneously via a stimulated process. The required momentum conservation leads to a change in the transverse momentum distribution of the electron. The matter-wave interaction in this case is mediated by the ponderomotive potential forming from the optical standing wave. In the classical force picture, the resulting ponderomotive force pushes the electron out of the high-intensity region leading to a change in the transverse momentum distribution of the electron beam. The experimental realization of the Kapitza-Dirac effect, nearly 70 years after its theoretical prediction^2,3^, and the interest in the quantum-coherent control of the electron wavepacket in ultrafast electron microscopes^7–13^ lead to a generalization of the Kapitza-Dirac effect in various scenarios. These include the expansion to the multiphoton regime of intense laser fields^14^, including relativistic corrections^15^, generalization to include two different wavelengths for light beams^16^, and the demonstration of quantum-path interferences in the Kapitza-Dirac scattering^17^. Beyond this elastic electron-light interaction, there has been an effort to achieve inelastic electron-light interaction in free space. Works in this direction proposed the generalizing of the Kapitza-Dirac effect to laser fields with two frequency components propagating along different directions, thereby realizing inelastic electron scattering from standing bichromatic^18^, and traveling bichromatic fields^10–12,19,20^. The second scheme utilized two inclined laser beams with different wavelengths that causes a dispersive propagation of the electron wavepacket in the potential landscape generated by the optical waves. This kind of interaction was recently demonstrated in two challenging experiments. The first experiment shows the possibility of accelerating the electron beam in vacuum with free-space light^10^, while the second experiment demonstrates the modulation of the longitudinal momentum of the electron wavepacket and the formation of an attosecond pulse train^12^. Emerging from the concept of an inelastic Kapitza-Dirac effect, Huang et al.^21^ proposed the possibility of creating non-Gaussian matter waves.

Photon-induced near-field electron microscopy (PINEM) is an alternative technique for the energy and momentum transfer between electron wavepackets and light^7,8,22^. PINEM is the result of the inelastic scattering of electrons from optical near fields, where the electrons experience a spectral modulation that shows symmetric quantized sideband peaks. This interaction opened up possibilities for attosecond control of free-electron quantum wavepackets^23^ and electron pulse manipulation^24^.

Altogether these works tried to overcome the gap in energy-momentum conservation for free-space electron-light interaction^22^. So far, all the considered inelastic free-space electron-light interactions leading to the PINEM-like effects and bunching of the electron wavepacket are requiring at least two waves at different frequencies and did not consider structured light, although inelastic electron scattering and temporal compression of the electron wavepacket, to reach femtosecond electron pulses with structured light, have been intensively studied^25–28^. In this letter we propose an interaction scheme where a structured light wave is used for achieving energy modulation of an electron wavepacket, resulting in a PINEM-like electron spectrum.

## Results and discussion

We study the propagation of an electron through a traveling time-harmonic electromagnetic light wave, which is represented by its vector potential $\vec{A}\left(\vec{r},t\right)$. The vector potential in this work is a time-harmonic transverse electromagnetic wave in the shape of a Hermite-Gaussian (HG) beam, a well-known exact solution of the free-space paraxial wave equation in Cartesian coordinates. The considered time-harmonic *x*-polarized HG beam that is propagating along the *y*-axis is given by^29^,

1 $$\begin{array}{c}{\vec{A}}_{n,m}(x,y,z;\omega )=\hat{x}\,{A}_{0}\frac{{w}_{0}}{W(y)}{H}_{n}\left(\frac{\sqrt{2}x}{W(y)}\right){H}_{m}\left(\frac{\sqrt{2}z}{W(y)}\right)\exp \left(-\frac{({x}^{2}+{z}^{2})}{W{(y)}^{2}}\right)\\ \,\times \,\exp \left(i\left[{k}_{{{{{{\rm{ph}}}}}}}y-(1+n+m)\arctan \frac{y}{{y}_{{{{{{\rm{r}}}}}}}}+\frac{{k}_{{{{{{\rm{ph}}}}}}}({x}^{2}+{z}^{2})}{2R(y)}\right]\right){e}^{-i\omega t},\end{array}$$

where $\omega =2\pi c/\lambda$ is the angular frequency of the light wave and *A*_0_ the amplitude of the vector potential. $W(y)={w}_{0}\sqrt{1+{(y/{y}_{r})}^{2}}$ denotes the in y-direction evolving beam waist with *w*_0_ and ${y}_{r}=\pi {w}_{0}^{2}{n}_{m}/\lambda$ as the beam waist and Rayleigh range respectively. $R(y)=y\,[1+{(y/{y}_{r})}^{2}]$ is the radius of curvature. *H*_*n*_ and *H*_*m*_ are the Hermite polynomials.

In this work we focus on the HG mode of the order HG_10_. Applying this in Eq. (1), we obtain the following equations for the vector potentials in two dimensions,

2 $$\begin{array}{c}{A}_{1,0}(x,y;\omega )={A}_{0}\frac{{w}_{0}}{W(y)}\frac{2\sqrt{2}x}{W(y)}\exp \left(-\frac{{x}^{2}}{W{(y)}^{2}}\right)\\ \times \,\exp \left(i\left[{k}_{{{\rm{ph}}}}y-(1+n)\arctan \frac{y}{{y}_{R}}+\frac{{k}_{{{\rm{ph}}}}{x}^{2}}{2R(y)}\right]\right){e}^{-i\omega t},\end{array}$$

and one dimension,

3 $${A}_{1,0}(x,y=0;\omega )={A}_{0}\frac{2\sqrt{2}x}{{w}_{0}}\exp \left(-\frac{{x}^{2}}{{w}_{0}^{2}}\right){e}^{-i\omega t}.$$

**Fig. 1:**
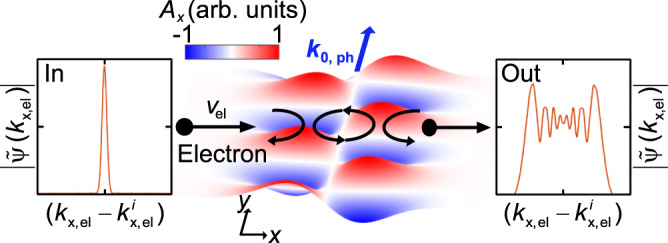
Inelastic electron scattering from a traveling Hermite-Gaussian optical beam. Inelastic scattering of a free-electron wavepacket with the center group velocity *v*_el_ at a traveling Hermite-Gaussian optical beam. The electron is scattered by the resulting Hermite-Gaussian shaped ponderomotive potential and experiences a self-interference phenomenon during this process, along the longitudinal direction, that leads to the modulation of the electron kinetic energy after the interaction.

**Fig. 2:**
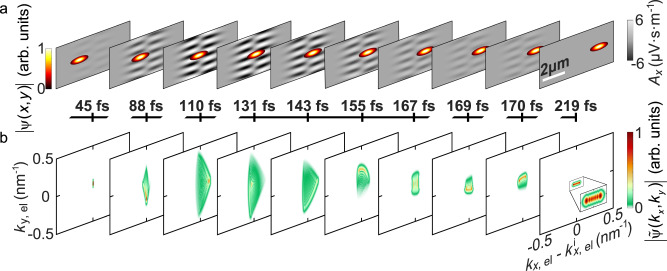
Interaction dynamics in spatial and momentum space. Dynamics of the evolution of a Gaussian electron wavepacket in the spatial and momentum space through a Hermite-Gaussian (HG_10_) pulsed laser beam (the laser electric-field-amplitude, its wavelength, and its temporal full width at half maximum (FWHM) are ${E}_{0}=15\,\times {10}^{9}\space{{{{{\rm{V}}}}}}{{{{{{\rm{m}}}}}}}^{-1}$, 700 nm and 28 fs, respectively) at different selected time steps. The electron wavepacket has an initial kinetic energy of 1 keV. The electron wavepacket has initial longitudinal and transverse broadenings (FWHM) of 250 nm and 60 nm, respectively. **a** The *x*-component of the vector potential representing the Hermite-Gaussian structured light field (gray background) at depicted time steps, with the insets demonstrating the amplitude of the electron wavepacket. **b** Electron momentum distribution at the corresponding time steps.

We consider an electron wavepacket $\psi \left(\vec{r},t\right)$ propagating through such a shaped laser field, experiencing a spatially varying ponderomotive potential along its trajectory on the *x*-axis (Fig. 1). The generation of time-harmonic shaped light pulses have been achieved with a variety of well-established techniques in a broad optical spectral range^30–33^. For understanding the physics of this system, we utilize a previously developed numerical toolbox that solves the time-dependent Schrödinger equation within the minimal-coupling Hamiltonian formalism^13,17,34^. This method allows for retrieving the interaction dynamics by capturing the modulation of the electron wavepacket and momentum-space probability amplitude $|\widetilde{\psi }(\vec{k})$| at each time step during the interaction. For this we define the transversal momentum ${k}_{y,{{{{{\rm{el}}}}}}}$ and longitudinal momentum ${k}_{x,{{{{{\rm{el}}}}}}}$. The electron parameters for controlling the strength of the interaction are the electron initial velocity (*v*_el_), longitudinal (*W*_L_) and transverse (*W*_T_) broadening (Full width at half maximum (FWHM)) of the electron wavepacket. We first investigate the propagation of a Gaussian electron wavepacket with ${W}_{{{\rm{L}}}}=250\,{{{{{\rm{nm}}}}}}$, ${W}_{{{\rm{T}}}}=60\,{{{{{\rm{nm}}}}}}$ and the center kinetic energy of 1 keV through a time-harmonic HG_10_ beam (see Eq. (2)) with central wavelength of $\lambda =700\,{{{{{\rm{nm}}}}}}$, temporal broadening (FWHM of the laser pulse) of 28 fs and the beam waist of ${w}_{0}=2\lambda$ (Fig. 2a). During the interaction with the HG_10_ beam, the phase of the electron wavepacket undergoes a wiggling motion, which is most prominently visualized in the momentum space (Fig. 2b). We observe distinct motions along both *x* and *y* directions, i.e., perpendicular and along the propagation direction of the optical beam, respectively. Within the time frame of 0–80 fs, the wiggling motion is most pronouncedly directed towards the positive and negative x axis. This oscillating motion of the electron beam within this time frame resembles the motion of the electron in a pure HG_00_ Gaussian beam, which allows the electron to occupy the higher order transverse momentum states, only spontaneously^35^. For the case of HG_00_ beam, this transverse phase modulation though is averaged out after the interaction and does not lead to a pure momentum modulation, neither in the longitudinal nor in the transverse direction. However, in the case of the HG_10_ beam considered here, this transverse wiggling motion is subsequently followed by a longitudinal oscillation within the time frame of 80–140 fs, when the electron travels within the low-intensity region of the optical beam, and thereafter again a wiggling motion along the transverse direction is occurred, until $t=180\,{{{{{\rm{fs}}}}}}$, when the electron leaves the interaction region (See Supplementary Movie 1 for a better visualization of the wiggling motion of the electron beam). The final longitudinal momentum spectrum of the electron shows a modulation in the shape of an energy comb. This energy comb reveals distinct sidebands for both positive and negative longitudinal momentums. This is an indicator for both energy loss and gain processes on the electron wavepacket during the interaction. The final transverse momentum remains unchanged during the interaction, as one would expect for a single-beam electron-light scattering experiment^35^. Thus, the intermediate time steps visualize the dynamics of the electron populating transversal and longitudinal momentum states, spontaneously. Thereby we observe an oscillation in the momentum state population of the electron. This oscillation alternates between transversal and longitudinal momentum state population. The remained populated momentum orders appear as a pattern of thin maximum and minimum fringes that reassemble an interference pattern. The final longitudinal momentum comb leads to a final energy modulation of the electron wavepacket and reassembles the PINEM spectrum observed for the interaction of electron wavepackets with the near-field light distributions and therefore showing a state population that is similar to a quantum walk^36^. The spacing between the observed sidebands cannot be explained through the absorbed and emitted photon energy or momentum recoil, leaving an open question for the physics behind this observed interaction.

### Inelastic electron scattering by light-pulse-generated ponderomotive potentials

**Fig. 3:**
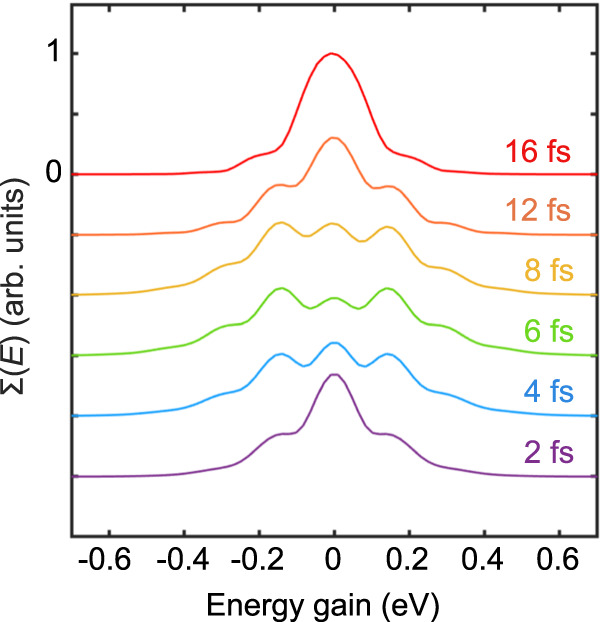
Dependence of inelastic scattering on the light pulse duration. Final electron energy-gain spectra $\Sigma (E)$ under the influence of a varied pulse duration (full width at half maximum (FWHM) in fs). The electron wavepacket has an initial carrier energy of 1.2 keV and longitudinal and transversal broadening of 150 nm and 60 nm respectively. The HG_10_ laser pulse electric-field-amplitude and wavelength are ${{{{{{\rm{E}}}}}}}_{0}=20\, \times {10}^{9}\space{{{{{\rm{V}}}}}}{{{{{{\rm{m}}}}}}}^{-1}$ and 200 nm, respectively.

The first observation of inelastic electron scattering from a single pulsed laser beam was made by Bucksbaum et al.^25^. This work attributes the observed energy exchange between the light field and the electron to the ponderomotive scattering from the temporal pulse envelope. As the pulsed laser beam scatters the electrons inelastically, the electrons acquire energy when scattered from the leading edge, but lose a similar amount of energy from the trailing edge. The energy exchange thereby depends on the pulse duration. However, only a significant and symmetric broadening of the electron wavepacket due to the inelastic scattering were observed, in contrast with the bunching effects we observe here. Our calculations so far considered a fixed laser pulse duration (FWHM). To investigate the effect of pulse duration on the inelastic interaction, we have varied this parameter as well. As depicted in Fig. 3, our findings clearly indicate a robust correlation between the observed inelastic scattering and the laser pulse duration (for the role of the wavelength and the pulse synchronization see Supplementary Note 1). Further the calculations reveal a correlation between the pulse duration and the strength of inelastic energy exchange, indicating that shorter pulses result in more intense exchange. It is worth noting that the formation of bunching is only noticeable when the pulse duration supports a strong interaction. Following a rather periodic dependence of the energy spectra on the temporal duration of the laser pulse and as well on the interaction time, further self-trapping of the electron wavepacket in the energy domain might be observable^37,38^. However, the position of these bunches in the final electron energy-gain spectra remains constant across different pulse durations. For very short pulses, the interaction strength is weakened, due to the insufficient interaction time between the electron wavepacket and the electromagnetic field. A comparison with calculations for an unstructured HG_00_ laser pulse (see Supplementary Note 2 and Supplementary Movie 2) though suggests that the observed interactions in this work can neither be solely explained by inelastic scattering of the electron wavepacket at the temporal envelope of the laser pulse, nor two-frequency ponderomotive interactions^10^.

### Inelastic electron-light scattering defined by electron self-interference

A known interaction scheme, arising from the ponderomotive force in single beam structured electromagnetic fields, was elaborated by Hilbert et al.^27^. They proposed that the ponderomotive force originating from a Laguerre-Gaussian beam, which can be approximated by a harmonic potential around its origin, acts as a temporal lens on the electron, by compressing the wavepacket. While this type of harmonic oscillation of the electron wavepacket in the potential landscape can be considered as inelastic and similar to our interaction geometry, the analytical discussion provided is insufficient to explain the observed modulation of the electron energy-gain spectrum reported here, within the parameter sets considered in our interaction scenario. Starting from the observation of the interference phenomena, and that electron-light interactions can be treated as an optical phase modulation of the electron wave function within the Volkov approximation^17^, we derive here an analytical expression for the estimation of maximum longitudinal momentum gain for the electron in the optical field. The spatial profile of the HG_10_ beam in direction of the electron propagation leads to a double barrier ponderomotive potential acting on the passing electron. The observed interference pattern indicates that the electron acquires a phase change induced by the ponderomotive potential creating a Fabry-Perot like geometry for the electron wavepacket—this created system is described visually in Fig. 1. The phase modulation the electron would experience in such a geometry is determined by $\Delta \phi =2{k}_{{HG}}d$. Here *d* is the distance between the reflecting potential planes and ${k}_{{HG}}$ is the maximally supported momentum component by the light interferometer. Further we consider that the phase change that a free electron acquires in an electromagnetic field, in the special case of eikonal approximation is given by the Volkov phase^39^ ${\varPhi }_{{{{{{\rm{V}}}}}}}=\tfrac{{e}^{2}}{2\hslash {m}_{0}}{\int }_{0}^{t}\mid\vec{A}{(\vec{r},\tau )}\mid^{2}d\tau$. When simplifying the problem within the 1D case along the *x*-direction, we can insert the vector potential of the time-harmonic HG_10_-beam as described by Eq. (3) into this phase expression and by further inserting it in the rearranged expression ${k}_{{{{{{\rm{HG}}}}}}}=\tfrac{{e}^{2}}{2\hslash {m}_{0}}\tfrac{1}{2d}{\int }_{0}^{t}\mid\vec{A}{(\vec{r},\tau )}\mid^{2}d\tau =\tfrac{{e}^{2}}{2\hslash {m}_{0}}\tfrac{4{A}_{0}^{2}}{{w}_{0}^{2}{v}_{{{{{{\rm{el}}}}}}}d}{\int }_{-\infty }^{\infty }{x}^{2}{{\rm{exp}}}(\tfrac{2{x}^{2}}{{w}_{0}^{2}})dx$ we obtain the following expression for the exchanged momentum:

4 $${k}_{{{{{{\rm{HG}}}}}}}=\frac{{e}^{2}}{\hslash {m}_{0}}\frac{{E}_{0}^{2}}{{\omega }^{2}{v}_{{{{{{\rm{el}}}}}}}}\frac{\sqrt{{{{{{\rm{\pi }}}}}}}}{4\sqrt{2}}\frac{{w}_{0}}{d}$$

**Fig. 4:**
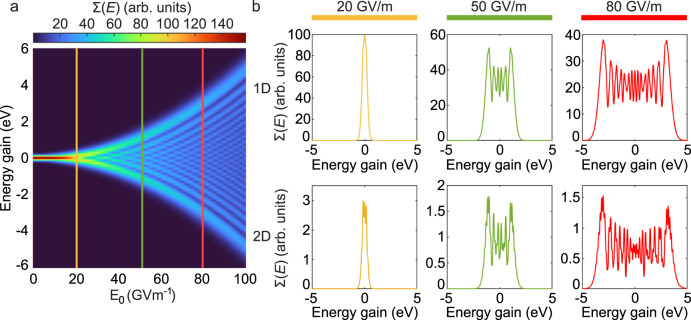
Field dependence of the inelastic scattering. The influence of the electric field amplitude *E*_0_ on the strength of the inelastic scattering. **a** Electron energy-gain spectrum Σ(*E*) versus the electric-field amplitude of the incident laser field (the laser wavelength, and its temporal full width at halfmaximum (FWHM) are 200 nm and 8 fs, respectively). The spectra were numerically calculated for a simplified one-dimensional (1D) system. **b** The comparison between 1D and two-dimensional (2D) systems at selected field strength of 20 GVm^−1^ (yellow), 50 GVm^−1^ (green), and 80 GVm^−1^ (red). The electron wavepacket has an initial center kinetic energy of 1.2 keV. The wavepacket has initial longitudinal and transverse broadenings (FWHM) (2D) of 150 nm and 60 nm, respectively.

**Fig. 5:**
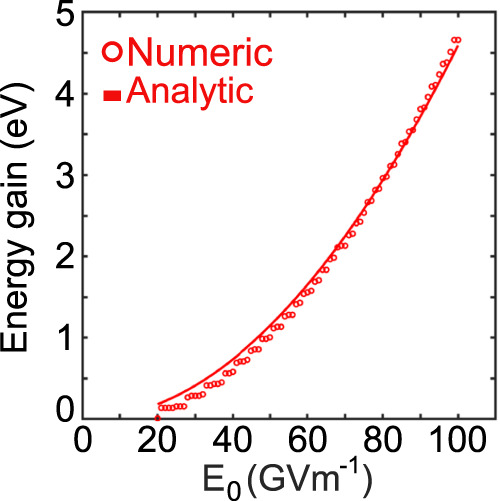
Analytical model describing the electron self-interference. Fit of the analytical model for the maximal energy exchange to the numerically obtained values from Fig. 4. The fitting parameter is *d* with *d* = 1083 nm.

Thereby we used ${{{{E}}}}_{0}={{{{{\rm{\omega }}}}}}{A}_{0}$ and related the time delay *τ* to the propagation distance *x* by $x={v}_{{{{{{\rm{el}}}}}}}\tau$ with *v*_el_ being the electron velocity. According to our proposed model we should expect a quadratic dependency from the electric field strength and an inverse proportionality from the electron velocity for the observed momentum exchange. Therefore, we further numerically studied the proposed system shown in Fig. 1 for different field strengths and electron velocities. Due to the large computational size for the system investigated in Fig. 2, we simplified the calculation by solving the Schrödinger equation for a 1D wave function in a 1D external vector potential. The applied algorithm for solving this problem stayed unchanged compared to the 2D solver. To verify these results, we compare the 1D calculation with 2D calculations for selected field strengths and electron velocities. For this we considered a time-harmonic HG_10_ beam with a 200 nm wavelength, temporal broadening of 8 fs and beam waist of ${w}_{0}=2{{{{{\rm{\lambda }}}}}}$. The electron wavepacket parameters were adjusted accordingly to ${W}_{L}=150{{\,{{{\rm{nm}}}}}}$ and ${W}_{T}=60{{{\,{{{\rm{nm}}}}}}}$. The investigated dependence on the electric field strength (Fig. 4) clearly shows a quadratic trend leading to an increased energy exchange for strong electric fields. For field strengths below 20 GVm^−1^, there is no modulation of the electron energy spectrum. Field strengths above 20 GVm^−1^ show the sidebands in the electron energy spectrum. These sidebands thereby seem to follow a quadratic trend with increased field strength. To review this observed trend, we retrieve the exchanged maximal momentum from Fig. 4a at each considered field value. These are then used to fit the expression for the momentum exchange (Eq. (4)) (see Fig. 5) with the distance *d* as the fit parameter. The resulting fit function (${d}_{{fit}}=1083\,{{{{{\rm{nm}}}}}}$) is in good agreement with the numerically calculated maximal momentum exchange. Further noticeable is that the obtained value of *d*, is close to the node to node distance ($d=900\,{{{{{\rm{nm}}}}}}$) between the peak amplitudes of the HG_10_ profile. The comparison between 1D and 2D calculations further support the observed trends, but indicate that the interaction in 2D leads to additional and more sharp sidebands (Fig. 4b). This is due to additionally available transverse momentum states in the 2D geometry. When reconsidering the dynamics of the interaction (Fig. 2b) we observe a gradual occupation of transverse momentum states. These transverse momentum states open up additional quantum paths for the electron wavepacket. These paths thereby can interfere with the direct longitudinal transition paths (see Supplementary Movie 1 and Supplementary Note 3), leading to additionally occupied momentum states in the 2D system.

**Fig. 6:**
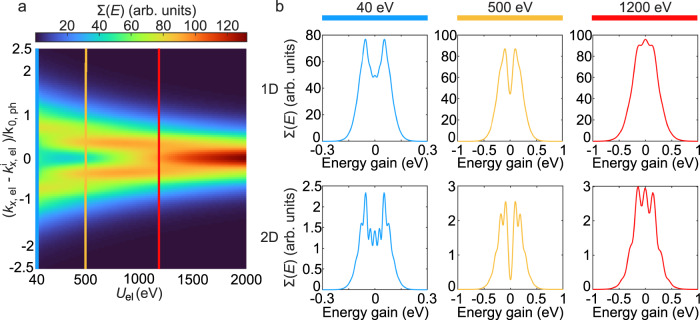
Electron kinetic energy dependence of the inelastic scattering. The influence of the electron kinetic energy *U*_el_ on the strength of the inelastic scattering. **a** The final electron energy-gain spectra Σ(*E*) versus the initial electron kinetic energy. The spectra were numerically calculated for a simplified 1D system. **b** The comparison between 1D and 2D systems at selected electron kinetic energies of 40 eV (blue), 500 eV (yellow), and 1200 eV (red). The considered Hermite-Gaussian light field has the electric-field-amplitude, wavelength, and temporal full width at halfmaximum (FWHM) of E_0_ = 20 × 10^9^ Vm^−1^, 200 nm and 8 fs, respectively. The wavepacket has initial longitudinal and transverse broadenings (FWHM) (for the 2D case) of 150 nm and 60 nm, respectively.

The strength of the momentum exchange versus the initial electron kinetic energy shows the expected inverse proportionality (Fig. 6). This means that by decreasing the electron kinetic energy, we observe an increase in the order of the momentum exchange. The enhanced momentum exchange at lower electron kinetic energies does not just affect the width of the energy-gain spectrum it also leads to the appearance and sharpening of two distinct sidebands. The 2D simulations again show additional sidebands. The investigation of the kinetic energy dependence further demonstrates the limitations of the proposed phenomenological model here for low electron kinetic energies. In this specific case we observe the upper interaction limit for electron kinetic energies of around 1.5 keV for a laser field with λ = 200 nm and ${E}_{0}=20\times {10}^{9}\,{{{{{\rm{V}}}}}}{{{{{\rm{m}}}}}}^{-1}$. Note that a slow electron also experiences a longer interaction time with the structured electromagnetic field. All together we conclude to have a good agreement between our numerical calculations of the maximum momentum exchange and the analytical estimate given by Eq. (2).

## Conclusion

In summary, our numerical calculations lead to a deeper understanding of free-space electron-light interaction in structured light fields. In this work the interaction of an electron wavepacket with a first-order Hermite-Gaussian laser pulse leads to the observation of a PINEM-like electron energy-gain spectrum by showing distinct sidebands. The exchange of longitudinal momentum is heavily influenced by both ponderomotive scattering from the laser pulse envelope and self-interference of the electron wavepacket. In addition, the interfering quantum paths in the transverse and longitudinal directions significantly impact the final momentum spectra. This interaction therefore demonstrates significant differences from so far known free-space inelastic electron-light interactions. Our studies further showed that the electron kinetic energy and the strength of the electric field are the key parameters in controlling the described interaction scheme. Furthermore, we were able to calculate the maximum momentum transfer based on a matter-wave Fabry-Perot model. We obtained these results using quantum mechanical calculations, but it’s worth noting that a classical explanation for the observed interaction might also be possible. Indeed, the existing understanding of electron-light scattering phenomena frequently encompasses both classical and quantum mechanical descriptions^10,19,37^.

The accuracy of our obtained calculations must be considered in light of the paraxial approximation utilized for the HG_10_ vector potential. This approximation, while suitable for many scenarios, loses accuracy when dealing with tightly focused beam experiments, as it fails to consider longitudinal field components that may alter the results^40,41^. To address this, we utilized a Maxwell-Schrödinger solver (details in ref. ^13^) to excite a higher-order Gaussian TM_10_ beam with an Hermite-Gaussian phase source in a finite-difference time-domain-based Maxwell solver. The resulting electron wavepacket exhibited a similar energy-gain spectrum as the calculations performed under the paraxial approximation (see Supplementary Note 4). We therefore conclude that our approximated calculations suffice for describing the main features in the interaction of the electron wavepacket with structured light fields. Hence, the longitudinal modulation of the electron wavepacket and the energy transfer from single-beam structured light to the electron wavepacket is valid for both cases.

For an experimental observation of the effect described here, the generation of high intense Hermite-Gaussian laser pulses is a key experimental parameter. The generation of intense Hermite-Gaussian pulses is realistic in today’s laboratory settings^30–33^. To further determine experimental feasibility, it is important to keep in mind that the calculations in this work consider a highly-coherent electron wavepacket with a spatial wavepacket broadening (FWHM) which is directly related to the electron energy spread and its pulse duration (see Supplementary Note 5). This assumption translates to an electron pulse of high temporal coherence, which essentially refers to the self-similarity of the electrons in the electron pulse. Consequently, a successful experiment would require a short electron pulse with high temporal coherence.

The state-of-the-art ultrafast scanning electron microscopy (USEM) setups^42,43^ are at the limit of this working parameters, by offering electron pulses with suiting electron energy spread but pulse durations of around 200 fs from photoemission tips. These pulses are known to have temporal coherence lengths of a few laser cycles^44^ but still have demonstrated the ability of coherent electron-light interactions. A more feasible candidate for demonstrating the self-interference effect in interaction with the structured light might be an experiment based on flat photoemission cathodes, that are known to produce single electron pulses with pulse duration below 100 fs^45^. Another approach would be the adjustment of the laser pulse parameters to longer pulse durations to overlap the available coherence lengths in ultrashort electron pulses. Hence, the visibility of the interference fringes described here emerges as a potential experimental method for assessing the temporal coherence characteristics of an electron beam, as it demonstrates a high sensitivity to the temporal coherence and synchronization between the electron and laser pulses. Besides the current achievements in coherent ultrashort electron pulse generation, the results of this work might get more accessible with future developments in ultrafast electron sources. Already today there is a variety of new concepts in electron field emission including single cooled atoms^46^, single-atom tip sources^47^, single-crystalline surfaces^48^, and superconducting niobium field emission tips^49^. Finally, it should be mentioned, that our results indicate that any structured light beam is feasible for this interaction when being able to support the self-interference of the electron wavepacket in the ponderomotive potential landscape of the interacting light beam. Feasible candidates might be Laguerre-Gaussian beams, Ince Gaussian beams and Bessel beams^50^. This variety of structured light fields as well as the application of this effect together with the Kapitza-Dirac effect might lead to full quantum control of the electron wavepacket via single structured light beams.

## Methods

### Time-dependent Maxwell-Schrödinger scheme^13,17,34^

For simulating the dynamics of the electron wavepacket, we have used the minimal-coupling Hamiltonian including the temporally and spatially varying vector potential. The time-dependent Schrödinger equation including the minimal-coupling Hamiltonian is given by:

5 $$i\hslash \frac{\partial }{\partial t}\psi (\overrightarrow{r},t)=\left[-\frac{{\hslash }^{2}}{2{m}_{0}}{\nabla }^{2}+\frac{{e}^{2}}{2{m}_{0}}{\left|\overrightarrow{A}(\overrightarrow{r},t)\right|}^{2}-\frac{i\hslash e}{{m}_{0}}\overrightarrow{A}(\overrightarrow{r},t)\cdot \overrightarrow{\nabla }\right]\psi (\overrightarrow{r},t).$$

Here we applied the coulomb gauge $\vec{\nabla }\cdot \vec{A}=0$ and $\psi (\vec{r},t)$ is the time-dependent electron wave function. Further, $\vec{A}(\vec{r},t)$ is the vectorpotential, *m*_0_ is the electron mass, ℏ is the reduced Planck’s constant, and *e* is the electron charge.

For the calculations used to obtain the results outlined in the manuscript Figs. 1–5 and Supplementary Note 1, 2, 5, and parts of Supplementary Note 4 the vectorpotentials were calculated analytically as the solutions to the paraxial Helmholtz equation and updated for each time step of the simulation accordingly with:

6 $$\begin{array}{c}{A}_{1,0}(x,y;\omega )={A}_{0}\frac{{w}_{0}}{W(y)}\frac{2\sqrt{2}x}{W(y)}\exp \left(-\frac{{x}^{2}}{W{(y)}^{2}}\right)\\ \times \exp \left(i\left[{k}_{{{{{{\rm{ph}}}}}}}y-(1+n)\arctan \frac{y}{{y}_{{{{{{\rm{r}}}}}}}}+\frac{{k}_{{{{{{\rm{ph}}}}}}}{x}^{2}}{2R(y)}\right]\right){e}^{-i\omega t}{e}^{-{(t/2\tau )}^{2}}.\end{array}$$

For the HG_10_ beam and with:

7 $$\begin{array}{c}{A}_{0,0}(x,y;\omega )={A}_{0}\frac{{w}_{0}}{W(y)}\exp \left(-\frac{{x}^{2}}{W{(y)}^{2}}\right)\\ \times \exp \left(i\left[{k}_{{{{{{\rm{ph}}}}}}}y-\arctan \frac{y}{{y}_{{{{{{\rm{r}}}}}}}}+\frac{{k}_{{{{{{\rm{ph}}}}}}}{x}^{2}}{2R(y)}\right]\right){e}^{-i\omega t}{e}^{-{(t/2\tau )}^{2}},\end{array}$$

for the HG_00_ beam. The variable *τ* denotes the FWHM of the laser pulse. Further terms are defined in the main text. The size of the discretization units of the spatial and temporal simulation domain are selected with $\delta x=\delta y=1.8\,{{{\rm{nm}}}}$ and $\delta t=0.4\delta x/c$ respectively.

The time propagator is approximated using a second-order differencing scheme, while spatial differentiation is achieved through consecutive steps of Fourier transformation, multiplication by appropriate transfer functions, and inverse Fourier transformation. This method, commonly referred to as the Fourier method, offers the advantage of stability and faster convergence compared to finite differentiation in molecular dynamics simulations^34,51^. Moreover, the accuracy of the results is validated by maintaining the norm of the wave function as close as possible to $N=\int {d}^{3}r|\psi (\vec{r},t)|$ at each given time. The overall longitudinal momentum distribution is given by an integration of the form $P({k}_{y})=\int d{k}_{x}|\widetilde{\psi }({k}_{x},{k}_{y})|$ for each time step. This can be related with energy momentum relation $E={\hbar }^{2}{k}_{y}^{2}/(2{m}_{0})$ to the overall electron energy-gain spectrum $\Sigma (E)$. The initial electron state is modeled by an analytical expression for a Gaussian wavepacket^34^:

8 $${\psi }_{0}(x,y,t=0)=\exp (i{k}_{x,{{{{{\rm{el}}}}}}}^{i}x){\left(\frac{1}{2\pi {W}_{{{{{{\rm{T}}}}}}}{W}_{{{{{{\rm{L}}}}}}}}\exp \left\{-\frac{1}{2}\frac{{(x-{x}_{0})}^{2}}{{W}_{{{{{{\rm{L}}}}}}}^{2}}\right\}\exp \left\{-\frac{1}{2}\frac{{(y-{y}_{0})}^{2}}{{W}_{{{{{{\rm{T}}}}}}}^{2}}\right\}\right)}^{\frac{1}{2}}.$$

For this *W*_*L*_ and *W*_*T*_ define the longitudinal and transverse broadening (Full width half maximum (FWHM)) of the electron wavepacket. The wavepacket is initially centered around (*x*_0_,*y*_0_) and has the initial electron momentum of ${k}_{{{{x}}},{{{\rm{el}}}}}^{(i)}={m}_{0}{v}_{{{{\rm{el}}}}}/\hbar$.

The solutions presented in Supplementary Note 4 demonstrate a comparison between calculations based on numerical solutions of Maxwell’s equations and the analytical approach discussed earlier. To obtain the numerical solution of Maxwell’s equations, we employed a finite difference time-domain method within a MATLAB environment using a custom-written numerical code^52^. The electromagnetic field was calculated in each time step and related to the vectorpotential with $\vec{B}=\vec{\nabla }\times \vec{A}$ and $\vec{E}={-\partial }_{t}\vec{A}$. The vector potentials from the Maxwell’s simulation domain were than mapped onto the Schrödinger simulation domain. Both domains were discretized using piecewise linear unit cells, although the size of the discretization units could vary. In Supplementary Note 4, we used $\delta x=\delta y=3\,{{{\rm{nm}}}}$ for the Maxwell domain, and $\delta x=\delta y=1.8\,{{{\rm{nm}}}}$ for the Schrödinger domain. Through our experience with this scheme, we have identified the limiting factors that influence the achievement of numerically convergent results. These factors include the Courant number of the finite-difference time-domain method (${S}_{c}=c\delta t/\delta x$) and the relative size of the unit cells in the simulation domains. Therefore, it is crucial to achieve a balance between accuracy and convergence to ensure reasonable simulation times.

### Supplementary information

Supplemental Material

Description of Additional Supplementary Files

Supplementary Movie 1

Supplementary Movie 2

## Data Availability

The Supplementary Movie 1 and Supplementary Movie 2 are supporting the conclusions of the paper. Additional data that support the findings presented in the main text and the Supplementary Information are available from the corresponding authors upon reasonable request.

## Code availability

The computer codes that support the findings presented in the main text and the Supplementary Information are available from the corresponding authors upon reasonable request.
