# Supplementary material for: Inelastic electron scattering at a single-beam structured light wave
Source: Commun Phys. 2023 Jul 15;6(1):179. doi: 10.1038/s42005-023-01300-2 (PMC11041727; doi:10.1038/s42005-023-01300-2)
Supplement: Supplementary file 1 — Supplemental Material [file 42005_2023_1300_MOESM1_ESM.docx]

**Inelastic Electron Scattering at a Single-Beam Structured Light Wave Supplementary Information**

**Sven Ebel^1,*^ and Nahid Talebi^1,2,*^**

*^1^Institute of Experimental and Applied Physics, Kiel University, 24098 Kiel, Germany*

*^2^Kiel Nano, Surface and Interface Science KiNSIS, Kiel University, 24118 Kiel, Germany*

E-Mail: talebi@physik.uni-kiel.de, ebel@physik.uni-kiel.de

**Supplementary contents:**

Supplementary Note 1: Laser Pulse Duration and Synchronization

Supplementary Note 2: Inelastic Electron scattering from a Single HG_00_ Beam

Supplementary Note 3: Quantum Paths in the 2D Hermite-Gaussian Beam

Supplementary Note 4: Calculations Beyond the Paraxial Approximation

Supplementary Note 5: Influence of the Wavepacket Dimension

**Supplementary Note 1: Laser Pulse Duration and Synchronization**

Given the strong correlation between the pulse duration and inelastic electron scattering, which is driven by energy exchange between the leading and trailing edges of the light pulse, it's reasonable to assume that the interaction is influenced by the synchronization between the arrival of the electron wavepacket and the light pulse. This can be demonstrated by simulating the interaction for different delay times between arrival of the electron wavepacket and the laser pulse. This dependence (Fig. S1 (a)) results in an asymmetric electron energy spectrum for longer delay times.


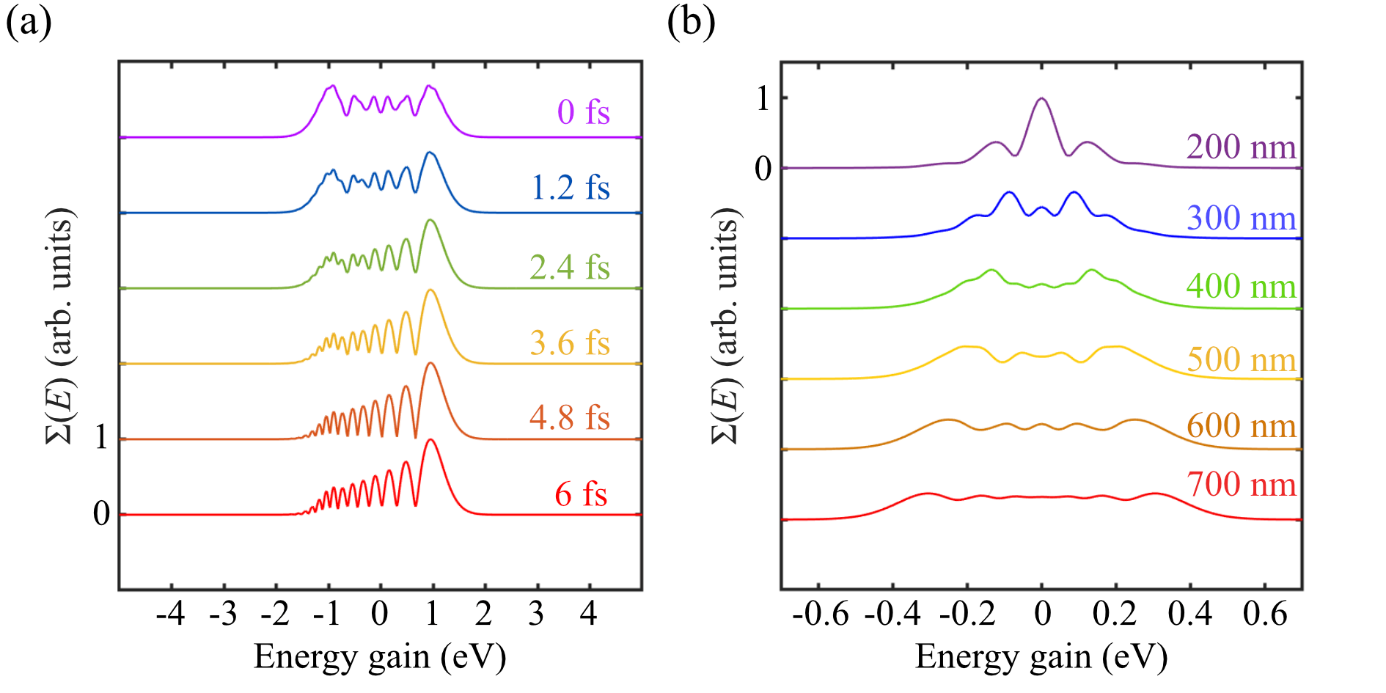


Fig. S1 (a) Final electron energy-gain spectra for a variation of laser pulse delay time. (b) Final electron energy-gain spectra for a variation of laser wavelengths simulated within the simplified 1D geometry. The electron wavepacket has an initial carrier energy of 1.2 keV and longitudinal broadening of 150nm. In (a) the HG_10_ laser pulse electric-field-amplitude and wavelength are $E_{0}=20 \times{10}^{9} V m^{-1}$ and 200 nm respectively. In (b) the HG_10_ laser pulse electric-field-amplitude and laser pulse duration is $E_{0}=20 \times{10}^{9} V m^{-1}$ and 2 fs respectively.

Finally, Fig. S1 (b) further highlights the fact that pulse duration is a variable for each selected parameter set. By keeping the pulse duration constant, we demonstrate that different interaction strengths can be achieved for varying laser beam wavelengths.

**Supplementary Note 2: Inelastic Electron scattering from a Single HG_00_ Beam**

To enhance our understanding of the electron-light interaction in a Hermite-Gaussian (HG_10_) pulsed laser beam, which is the focus of our work, we will outline a discussion of the electron-light interaction within a Gaussian (HG_00_) pulsed laser beam (Fig. S2). Therefore, we first compare the interaction dynamics for the HG_00_ beam with those already featured in the Fig. 2 of the manuscript for the HG_10_ beam. The simulation parameters for the HG_00_ simulation are chosen similar to those presented in Fig. 2. The initial electron energy is 1 keV. The electric field amplitude of the HG_00_ beam is $E_{0} = 5 \times{10}^{9} Vm^{-1}$, the wavelength is 700 nm and the pulse has a FWHM of 28 fs. The electron wavepacket has initial longitudinal and transverse broadenings of 250 nm and 60 nm, respectively (Fig S2 (a)).


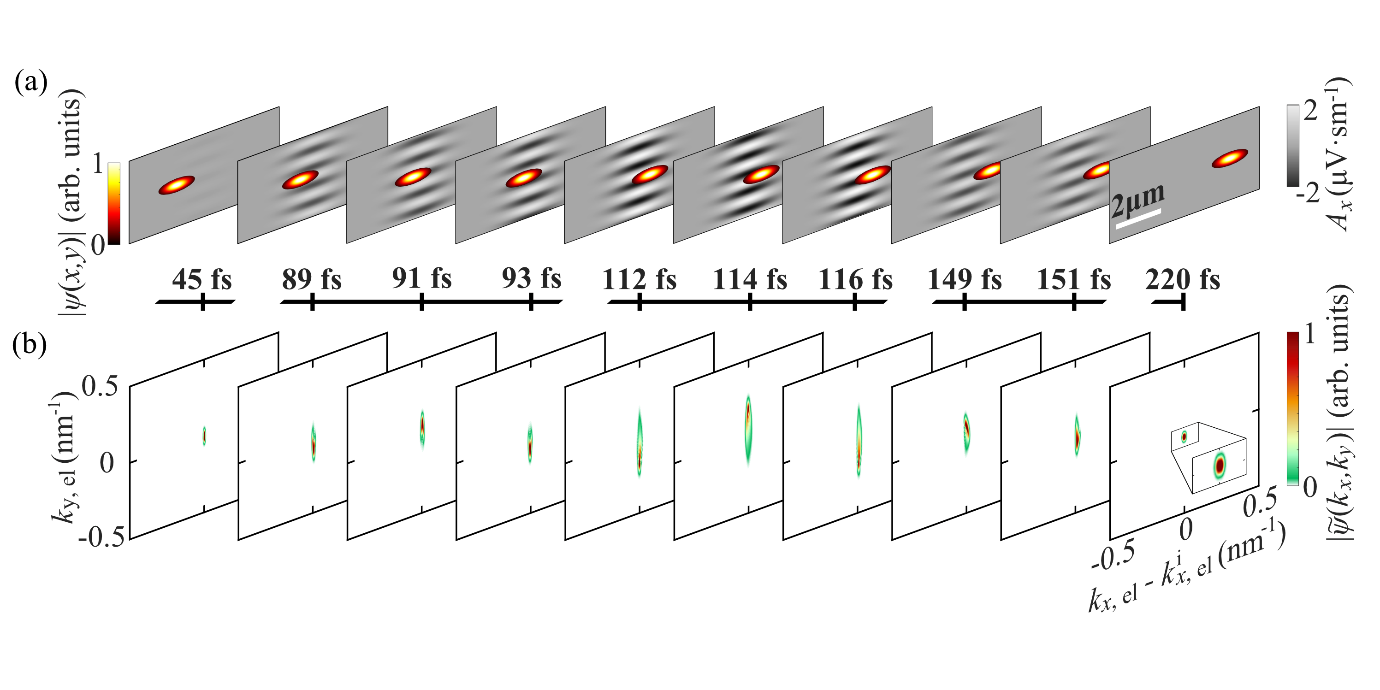


Fig. S2 Dynamics of the evolution of a Gaussian electron wavepacket in the spatial and momentum space through a Gaussian (HG_00_) pulsed laser beam (the laser electric-field-amplitude, its wavelength, and its temporal FWHM are $E_{0} = 5 \times{10}^{9} Vm^{-1}$, 700 nm and 28 fs, respectively) at different selected time steps. The electron wavepacket has an initial carrier energy of 1 keV. The electron wavepacket has initial longitudinal and transverse broadenings of 250 nm and 60 nm, respectively. (a) The *x*-component of the vector potential representing the Gaussian structured light field (gray background) at depicted time steps, with the insets demonstrating the amplitude of the electron wavepacket. (b) Electron momentum distribution at the corresponding time steps.

During the electron-light interaction, the momentum distribution of the electron displays phase-cycling behavior during the temporal population of transverse momentum states (Fig. S2 (b)). This manifest itself as an oscillatory population of higher-order transverse momentum states, peaking at the light pulse intensity apex and returning to the initial state as the electron exits the interaction zone. However, the interaction does not cause any oscillatory longitudinal momentum state population, though the longitudinal momentum spread in the electron distribution steadily increases, mainly due to the dispersive propagation of an electron pulse in vacuum [1]. In addition, the final electron longitudinal momentum population does not exhibit any bunching but shows momentum gain and loss after the interaction. This change in the longitudinal momentum of an electron was first observed in 1987 by Bucksbaum et al. [2] by studying the inelastic scattering of free electrons from a Gaussian (HG_00_) pulsed laser beam. They attributed the observed energy exchange between the light field and the electron to the ponderomotive scattering from the temporal pulse envelope. Fig. S3 shows further studies on the properties of this scattering mechanism. The inelastic scattering process thereby reaches its maximum momentum exchange for shorter laser pulse durations (Fig. S3 (a)). For too short pulse durations the energy-gain decreases again. This can be attributed to short interaction times between the electron and the laser pulse. The electron kinetic energy dependence of this scattering mechanism appears to be minor (Fig. S3 (b)). This contrasts with the strong anti-proportional initial electron kinetic energy dependence observed for an HG_10_ beam (e.g. Fig. 6). Fig. S3 (c) illustrates the difference in the final electron energy-gain spectrum for a HG_10_ and HG_00_ beam.


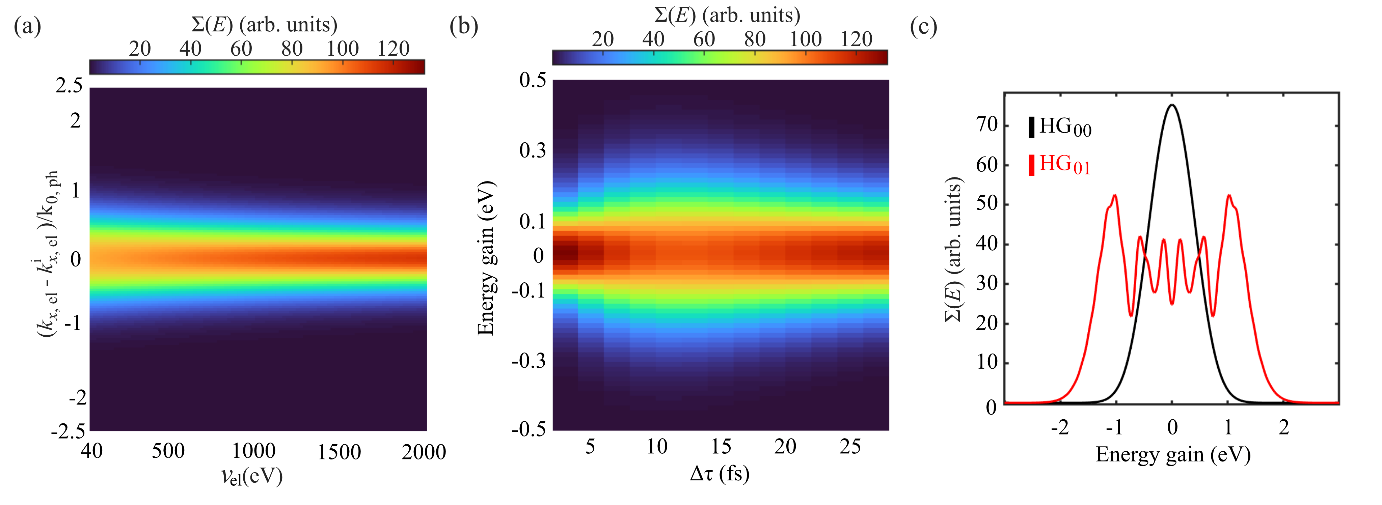


Fig. S3 The final electron energy-gain spectra versus the (a) initial electron kinetic energy and (b) laser pulse duration (FWHM in femtoseconds). (c) Comparison between the final electron energy-gain spectra after scattering of a HG_10_ (red) and HG_00_ (black) pulse. The spectra were numerically calculated for a simplified 1D system (a-c). The considered Hermite-Gaussian light field has the electric-field-amplitude, wavelength, and temporal broadening of $E_{0} = 20 \times{10}^{9} Vm^{-1}$ (a and b) or $E_{0} = 50 \times{10}^{9} Vm^{-1}$ (c), 200 nm and 8 fs, respectively. The wavepacket has initial longitudinal broadening of 150 nm. For the calculations in (b) and (c) the initial carrier energy was 1.2 kev.

**Supplementary Note 3: Quantum Paths in the 2D Hermite-Gaussian Beam**

Here we provide an illustration of the accessible quantum paths during the inelastic electron scattering by a structured light beam. The phase cycling of the electron wavepacket during the interaction into transverse momentum states opens up additional quantum paths for the electron wavepacket (Fig. S4). These paths thereby can interfere with the direct longitudinal transition paths, leading to additionally occupied momentum states.


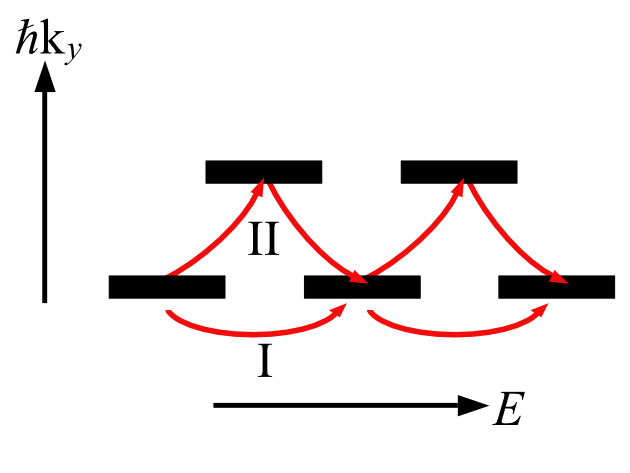


Fig. S4 The schematic of the interfering possible quantum paths in the energy-momentum diagram accessible in the 2D system. The arrows indicate the possible direct energy-state transitions (I) and interfering quantum paths (II).

**Supplementary Note 4: Calculations Beyond the Paraxial Approximation**

The calculations presented so far are all done within the paraxial approximation. The paraxial approximation is just valid for a small enough beam divergence and does not consider any longitudinal field components. It is known that especially the longitudinal field components can have an influence on the energy and momentum exchange between electron wavepackets and light [3,4]. This motivates further studies considering the influence of exact field solutions on the effects described in this work. Therefore, we utilize a Maxwell-Schrödinger solver (see [5]) where we excite a higher-order Gaussian TM_10_ beam with a Hermite-Gaussian Huygens source embedded in a finite-difference time-domain-based Maxwell solver. We consider an electron with longitudinal and transvers FWHM of 250 nm and 60 nm. The field intensity is set to 5 GVm^-1^ and the laser pulse has a wavelength of 500 nm with a temporal broadening of 20 fs at FWHM (Fig. S5 (a)).


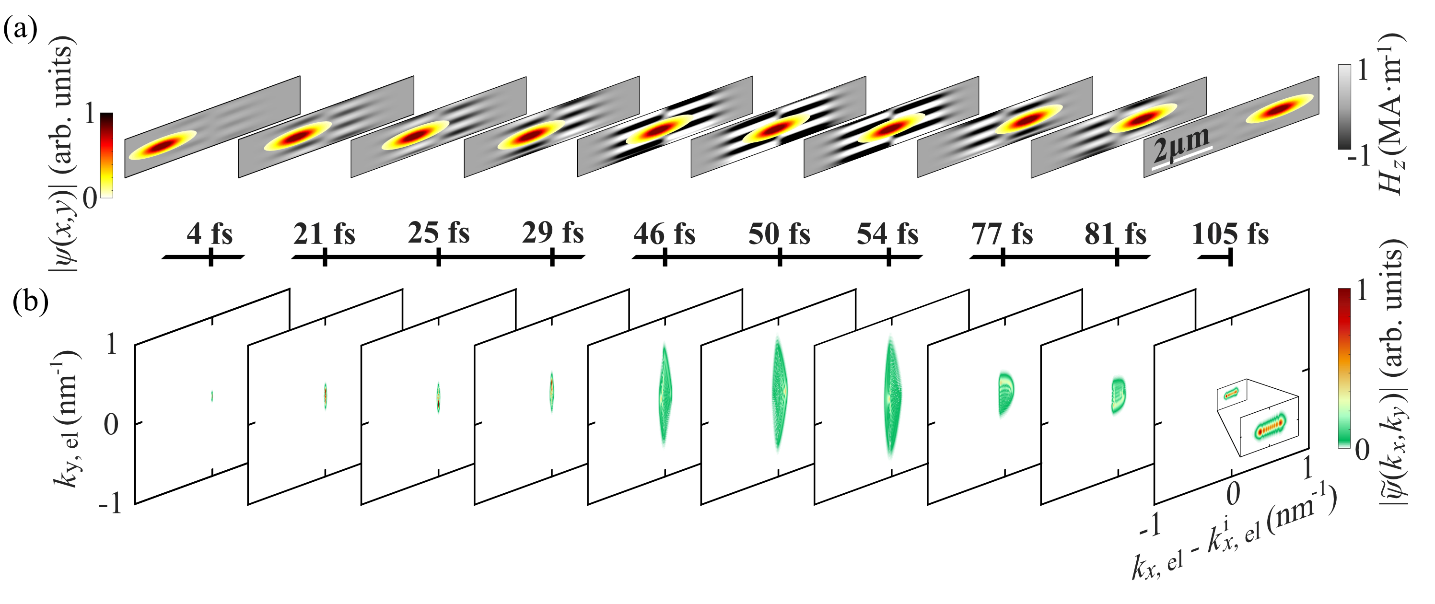


Fig. S5 Dynamics of the evolution of a Gaussian electron wavepacket in the spatial and momentum spaces propagating through a Maxwell-propagated Hermite-Gaussian (HG_10_) pulsed laser beam (the laser electric-field-amplitude, its wavelength, and its temporal FWHM are $E_{0} = 5 \times{10}^{9} Vm^{-1}$, 500 nm and 20 fs, respectively) at different selected time steps. The electron wavepacket has an initial kinetic energy of 1 keV and longitudinal and transverse broadenings of 250 nm and 60 nm, respectively. (a) The *z*-component of the magnetic field (gray background) at depicted time steps, with the insets demonstrating the amplitude of the electron wave function in the real space. (b) The distribution of the electron wavepacket in the momentum space at the corresponding time steps.

The resulting interaction is similar to those observed within the paraxial approximation (e.g. Fig. 2). Both the momentous population of longitudinal and transversal momentum states of the electron wavepacket are observable as well as the oscillation behavior within the population mechanism (Fig. S5 (b)). The final longitudinal momentum spectrum of the electron shows the formation of the characteristic bunch structure. The comparison between the Maxwell-Schrödinger and the resulting spectra from paraxially based calculations show a similar shape (see Fig. S6). Although the electron energy-gain spectra exhibit similar shapes with these parameters, the structure of the formed bunches differs between the Maxwell-Schrödinger and paraxial simulations. For this comparison the electromagnetic field in the paraxial approximation-based calculation has a field amplitude of 12 $\mathrm{GV}m^{-1}$ while the Source in the Maxwell solver is set to field intensities of 5 $\mathrm{GV}m^{-1}$. While the electron energy-gain spectra share similar shapes under these parameters, the Maxwell-Schrödinger and paraxial simulations result in different formed bunch structures. As it is challenging to precisely estimate the field strength that the electron wavepacket experiences in the Maxwell simulation, the impact of the different utilized field intensities cannot be solely explained by longitudinal field components. Nonetheless, we maintain that the differences between the two simulations are minor and that our manuscript's calculations provide an adequate portrayal of the observed phenomena.


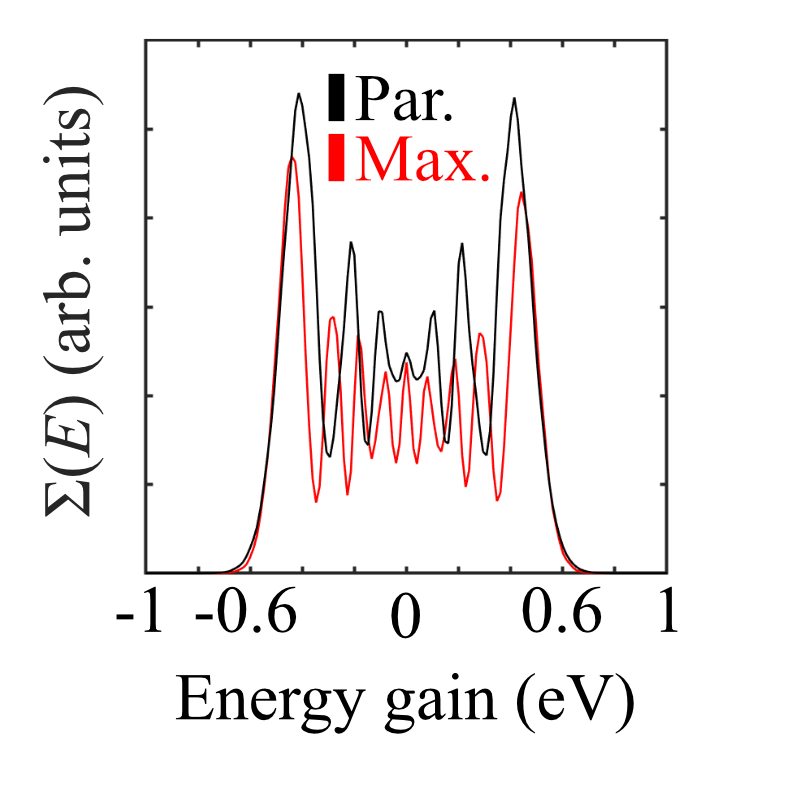


Fig. S6 Comparison between the electron energy-gain spectra simulated with the Maxwell-Schrödinger calculations (red) and with the paraxial approximation-based simulations (black). The electron wavepacket has an initial kinetic energy of 1 keV and has initial longitudinal and transverse broadenings of 250 nm and 60 nm, respectively in both calculations. The considered HG_10_ laser pulse has a wavelength and temporal broadening of 500 nm and 20 fs respectively in both calculations. The peak field intensity is set to 5 $\mathrm{GV}m^{-1}$ in the Maxwell-Solver and to 12 $\mathrm{GV}m^{-1}$ for the paraxial vector potential.

**Supplementary Note 5: Influence of the Wavepacket Dimension**

Here we provide a concise additional discussion on the influence of the dimensions of the electron wavepackets on the inelastic scattering of the electron wavepacket at the HG_10_ structured light beam. Our simulation shows that the electron wavepacket should be long enough to support the self-interference mechanism as demonstrated in Fig. S7 Therefore, a large spatial spread of the electron wavepacket is desired to ensure a longer interaction time – particularly, the spatial distribution of the electron wavepacket should be comparable or larger than the distance between the two lobes of the ${HG}_{10}$ light beam. The broadening of the transverse wavepacket, however, does not impact the overall energy exchange between the structured light wave and the electron wavepacket. Nonetheless, as depicted in Fig. S8, selecting a narrower wavepacket distribution in the transverse direction leads to a higher population of longitudinal momentum states.


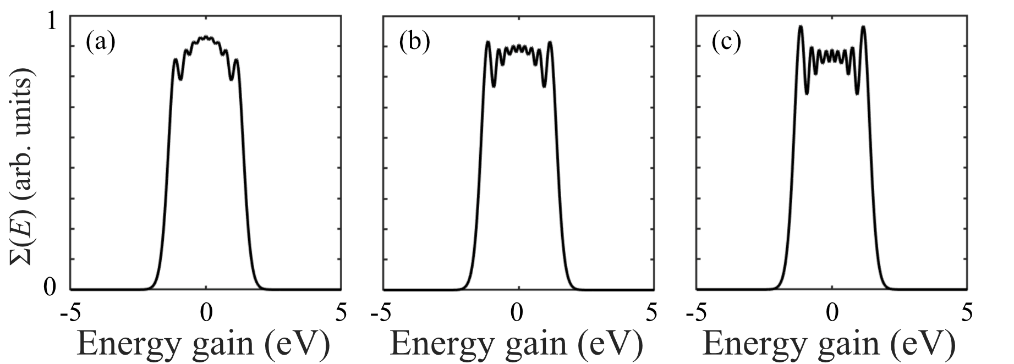


FIG. S7 The influence of the longitudinal broadening of the electron wavepacket on the final electron energy-gain spectra after the interaction. The depicted spectra correspond to longitudinal wavepacket broadening of (a) $W_{L}=130 \text{nm}$, (b) $W_{L}=140\text{ nm}$, and (c) $W_{L}=150 \text{nm}$. These spectra where numerically calculated for a simplified 1D geometry. The considered light wave is a Hermite-Gaussian (${HG}_{10}$) pulsed laser beam (Laser electric-field-amplitude, wavelength, and temporal broadening are $E_{0}=50 \times{10}^{9} V m^{-1}$, 200 nm and 8 fs, respectively). The considered electron wavepacket has an initial kinetic energy of 1.2 keV.


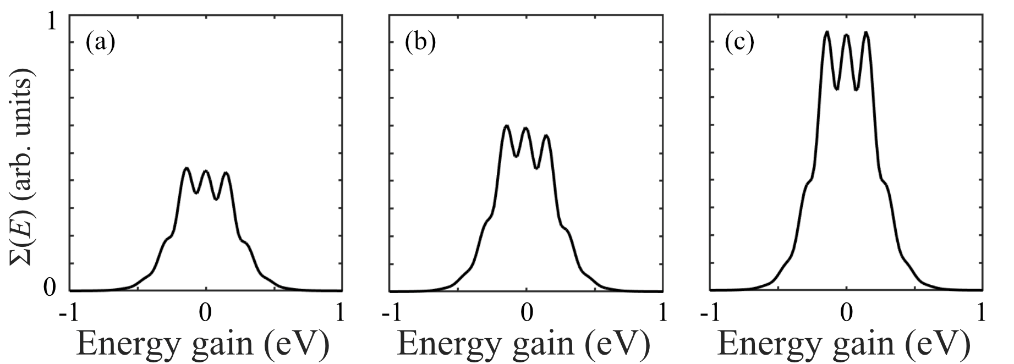


FIG. S8 The influence of the transverse broadening of the electron wavepacket on the final electron energy-gain spectra after the interaction. The depicted spectra correspond to transverse wavepacket broadenings of (a) *W*_T_ = 100 nm, (b) *W*_T_ = 60 nm, and (c) *W*_T_ = 20 nm. These spectra where numerically calculated for a 2D geometry. The considered Light wave is a Hermite-Gaussian (HG_10_) pulsed laser beam (Laser electric-field-amplitude, wavelength, and temporal broadening are $E_{0}=20 \times{10}^{9} V m^{-1}$, 200 nm and 8 fs, respectively). The considered electron has an initial kinetic energy of 1.2 keV and a longitudinal broadening of *W*_L_ = 150 nm.

**Supplementary References**

[1] N. Talebi, Schrödinger electrons interacting with optical gratings: quantum mechanical study of the inverse Smith-Purcell effect, New J. Phys. **18**, 123006 (2016).

[2] P.H. Bucksbaum, M. Bashkansky and T. J. McIlrath, Scattering of electrons by intense coherent light, Phys. Rev. Lett. **58**, 349 (1987).

[3] L. Cicchitelli, H. Hora and R. Postle, Longitudinal field components for laser beams in vacuum, Phys. Rev. A **41**, 3727 (1990).

[4] B.Quesnel and P. Mora, Theory and simulation of the interaction of ultraintense laser pulses with electrons in vacuum, Phys. Rev. E **58**, 3719 (1998).

[5] N. Talebi, Strong interaction of slow electrons with near-field light visited from first principles, Phys. Rev. Lett. **125**, 080401 (2020).
