# Supplementary material for: Inelastic electron scattering at a single-beam structured light wave
Source: Commun Phys. 2023 Jul 15;6(1):179. doi: 10.1038/s42005-023-01300-2 (PMC11041727; doi:10.1038/s42005-023-01300-2)
Supplement: Supplementary file 2 — Description of Additional Supplementary Files [file 42005_2023_1300_MOESM2_ESM.pdf]

Description of Additional Supplementary Files

File name: Supplementary Movie 1

Description:

Dynamics of an electron wavepacket in a Hermite-Gaussian shaped time-harmonic electromagnetic field. The considered light wave is a Hermite-Gaussian (HG_{10}) pulsed laser beam (laser electric-field-amplitude, wavelength, and temporal broadening are $E_0 = 50 \times 10^9\ \mathrm{V\,m^{-1}}$, 700 nm and 8 fs, respectively). The considered electron wavepacket has an initial kinetic energy of 1.0 keV. Longitudinal and transverse broadening are $W_\mathrm{L} = 250\ \mathrm{nm}$ and $W_\mathrm{T} = 60\ \mathrm{nm}$ respectively.

File name: Supplementary Movie 2

Description:

Dynamics of an electron wavepacket in a Gaussian shaped time-harmonic electromagnetic field. The considered light wave is a Gaussian (HG_{00}) pulsed laser beam (laser electric-field-amplitude, wavelength, and temporal broadening are $E_0 = 5 \times 10^9\ \mathrm{V\,m^{-1}}$, 700 nm and 8 fs, respectively). The considered electron wavepacket has an initial kinetic energy of 1.0 keV. Longitudinal and transverse broadening are $W_\mathrm{L} = 250\ \mathrm{nm}$ and $W_\mathrm{T} = 60\ \mathrm{nm}$ respectively.
